# The Impact of Knowledge of Suicide Prevention and Work Experience among Clinical Staff on Attitudes towards Working with Suicidal Patients and Suicide Prevention

**DOI:** 10.3390/ijerph13020195

**Published:** 2016-02-04

**Authors:** Inga-Lill Ramberg, Maria Anna Di Lucca, Gergö Hadlaczky

**Affiliations:** 1 National Center for Suicide Research and Prevention of Mental Health, NASP, Karolinska Institutet, Solna 171 77, Sweden; gergo.hadlaczki@ki.se; 2 Medical Management Centre, Department of Learning, Informatics, Management and Ethics, Karolinska Institutet, Solna 171 77, Sweden; maria.di.lucca@ki.se

**Keywords:** suicide prevention, attitudes, training, psychiatric staff, job clarity, job confidence, LISREL, regression

## Abstract

Suicide-preventive training has shown to influence attitudes. This study aimed at investigating what impact other factors than knowledge might have on attitudes towards work with suicidal patients and suicide prevention. In 2007, 500 health-care staff working in a psychiatric clinic in Stockholm received a questionnaire with items concerning work with suicidal patients to which 358 (71.6%) responded. A set of attitude items were tested using structural equation modelling (LISREL). Three models were found to be satisfactory valid and reliable: *Job clarity*, *Job confidence* and *Attitudes towards prevention*. These were then used in regression analyses as dependent variables with predictors such as *experience of work with suicidal patients, perceived sufficient training,* *age* and *gender*. *Perceived sufficient training* was consistently the most important predictor for all three attitude concepts (*p* < 0.01, β = 0.559 for *Job clarity*; *p* < 0.01, β = 0.53 for *Job confidence*; *p* < 0.01, β = 0.191 for *Attitudes towards prevention*). *Age* was another significant predictor for *Job clarity* (*p* < 0.05, β = 0.134), as was *experience of patient suicide* for *Job confidence* (*p* < 0.05, β = 0.137). It is concluded that providing suicide preventive education is likely to improve attitudes towards the prevention of suicide, clarity and confidence regarding their role in the care for suicidal patients. These improvements may contribute to the prevention of suicide in health care settings.

## 1. Introduction

According to the largest systematic review of suicide prevention interventions, the training of healthcare professionals in suicide prevention is an effective strategy [1]. Knowledge of how to identify, and manage suicidal patients is an important issue in suicide prevention [2]. Despite recommendations from clinical practice guidelines that emphasize, for instance, the importance of exploring suicidal thoughts and plans of depressed patients [3,4], there is a wide variation in physicians’ willingness to do so [5,6,7]. Training health professionals may increase the likelihood of guideline adherence, and in a number of studies, have been shown to have had positive suicide preventive effects (for reviews see [1,2,8]). This has been shown worldwide for different target groups with a range of different procedures and curriculums aimed at the prevention of suicide, where activities often revolve around increasing awareness and recognition of warning signs of suicide, improving adherence to recommended treatment-practices of associated mental health problems such as depression [9,10,11]. It is likely that these types of educational activities, not only increase the level of knowledge among health-care professionals but also improve attitudes (especially when the negative attitudes stem from a lack of knowledge about suicide prevention). The training of mental health professionals is, with a few exceptions [9,12,13,14], usually performed in small groups or in settings that make it difficult—not to say impossible—to measure direct effects on suicidal behavior [8]. For this reason many studies aiming to evaluate the effects of training in suicide prevention use other outcome measures, believed to have an intermediary effect on suicidality. Measures relating to attitudes regarding whether suicide is preventable, positive or negative views on working with suicidal patients, as well as professionals’ confidence in their ability in working preventively are common. These outcome measures are based on the belief that the distress of a suicidal individual can be counterbalanced by positive attitudes thus having a preventive effect on suicidal acts [15,16,17,18,19,20,21], although there are few studies that have systematically examined whether improving attitudes lead to an actual decrease in suicides (this is partly because improved attitudes are, in most cases, the result of increased knowledge, and the suicide preventive effects of the two are difficult to separate). In fact, the attitude-behavior link is one of the more controversial topics in the attitudes literature, due to both the complexity of attitudes and the difficulty in linking general attitudes with specific behaviors [22]. Evaluations of suicide-preventive training have also shown that the obtained attitude-change was not consistent over time [23,24,25]. Thus, there are many difficulties in using attitudes as an outcome measure in evaluating training in suicide prevention. However, one step forward would be to find and agree on adequate and valid measurements.

Attitudes refer to psychological processes that determine individual behavior [26,27,28,29,30,31], implying that attitudes cannot be observed directly and only inferred indirectly from observable indicators, which makes their measurement difficult. Another problem in using attitudes to evaluate the effectiveness of training in suicide prevention is that there is no overall agreement on how and with what they should be measured, although suicidologists for many years have used attitudes as an outcome measure [32,33,34,35,36,37,38,39,40,41]. Instead different approaches, measurement procedures and instruments have been used. One common approach is the use of questionnaires with attitude items, but their focus vary from the view on suicide as a phenomenon and a general view on the prevention of suicide [32,33,34,35,40,42,43] to attitudes towards caring for fictious cases [44,45,46]. Efforts have also been made to find more adequate and reliable attitude items to measure the effects of suicide-preventive training [38,39,40,41,47,48], with scales focusing on the ability to handle client situations, confidence in risk management and in work with suicidal patients and suicide risk management. Two Swedish studies focus on items related to psychiatric staff’s attitudes towards suicidal patients and towards their care [37,46,49]. 

As mentioned before, factors concerning the quality of care are believed to be crucial for the possibility to prevent suicidal behavior. Suicide-preventive care programs [50,51,52] are used in many health-care settings as suicidal patients’ life-threatening behavior necessitates clear-cut routines, which must be known and understood by all those who work with them. Many of the quality aspects are included in recent efforts to find adequate, valid and reliable measurement models to evaluate the effect of training in suicide prevention on attitudes. However, we find that one important perspective is still missing, namely how those who are working with suicidal patients perceive that they are supported by the organization. 

Although several international as well as Swedish studies have shown that attitudes can be changed through training in suicide prevention [34,36,37,39,40,41,43,48,53,54,55] they are also considered as being relatively stable [30,56], age or predispositions such as gender may have an important effect on attitudes, alongside prior experience of dealing with suicidal persons, as attitudes are expected to change as a function of experience.

The aim of this study is to investigate what impact age, gender, the experience of working with suicidal patients and the perceived level of training in suicide prevention have on attitudes towards the preventability of suicide and working with suicidal patients.

## 2. Method 

### 2.1. Participants

In 2007, mandatory training activities in suicide prevention were held in a psychiatric clinic in the southern region of Stockholm (South Psychiatry Stockholm) with a catchment area corresponding to 231,000 adults. Prior to the training all mental health-care staff (500 individuals) were invited to complete a questionnaire with items concerning work with suicidal patients. The training program as well as the evaluation results will be presented in a separate paper.

The number of participants who completed the questionnaire was 358 (71.6%) of which 38.3% were males and 61.7% were females and the mean age was 49.96. The participants were of different medical backgrounds such as psychiatrists (4.8%), doctors under training (3.6%), case managers (47.9%), contact persons (30.8%) and others such as psychologists, physical therapists and occupational therapists (12.9%). The case-managers were mainly psychiatric nurses with additional academic training in case management and the contact persons, mainly assistant nurses, with at least upper secondary school education specifically related to healthcare. 

### 2.2. Data Analysis

#### 2.2.1. Outcome Variables

There were three different dependent variables in this study. *Job clarity* was measured by 4 items [57,58] relating to subjects’ perception of whether they understood their tasks regarding work with suicidal patients. *Job confidence* was also measured by four items concerning subjects’ confidence regarding work with suicidal patients. *Attitudes towards prevention* constituted 4 items inquiring about subjects’ perception on whether suicide is preventable [49]. The four item scales were aggregated for the main analyses. These models were used in the regression.

There were six single-item independent variables used in the study. *Work experience* inquired about the number of years spent working in psychiatry, *Experience of work with suicidal patients* regarded whether subjects were exposed to suicidal patients in the past 6 months; *Experience of patient suicide* asked whether subjects had contact with patients who subsequently died by suicide; *Perceived sufficient training* was an item related to subjects’ perception of whether they had received sufficient training in suicide prevention; finally age and gender were inserted in the model. 

#### 2.2.2. Structural Equation Modelling 

Cronbach Alpha is used to test the reliability of many of the attitude scales that have been referred to in this paper (for instance [36,39,41]). But Cronbach Alpha tends to be sensitive to the number of items in the scale, the more items the higher probability that the Cronbach Alpha reaches an acceptable value, and it gives no information on the dimensionality of the concepts or details about the individual indicators in terms of validity and reliability. Therefore, a different approach was used in this study. The items concerning attitudes towards work with suicidal patients as well as the perceived possibility to prevent a suicidal act were investigated using LISREL 8.51 [59,60]. The application of LISREL in this context consists of specifying a confirmatory factor analysis model, testing the model-data consistency and giving information about the dimensionality of measurement instrument and the validity and reliability of the indicators. Confirmatory factor analysis has several advantages over exploratory factor analysis, which often produces results which are difficult to interpret and interpretations vary between researchers [49]. In contrast to exploratory factor analysis, a confirmatory factor analysis starts with a conceptual specification of a measurement model, thus, one have to be explicit about the measurement hypotheses. 

The models tested are the concepts of clarity in work with suicidal patients (*Job clarity*), confidence in work with suicidal patients (*Job confidence)* and possibilities to prevent suicidal acts (*Attitudes towards prevention*).

##### Job Clarity

Five items with four response alternatives from ”Do not agree at all” to ”Agree completely” previously used in Swedish studies [57,58] were aimed at measuring clarity in work with suicidal patients (*Job clarity*).

- I know what is expected of me in work with suicidal patients (KNOW)
- I get clear and good instructions concerning management of care of suicidal patients (CLEAR)
- Different superiors have varying views on how and what I shall do in work with suicidal patients (DIFFVIEW)
- Having irreconcilable demands made on me by different people in the ward (IRREDEM)
- I lack knowledge and information about what is important in work with suicidal patients (LACKINFO)

##### Job Confidence

Four items with four response alternatives from ”Do not agree at all” to ”Agree completely” were designed to measure confidence in work with suicidal patients. The items were designed in cooperation with the trainers in order to find indicators that measure how well some of the new routines were implemented.

- The division of responsibilities for risk assessment is clear and distinct (CLEARRES)
- I feel confident when working with suicidal patients (CONF)
- I have no one with whom I can share the responsibility for the suicidal patients (ALLRESP)
- The co-operation concerning the suicidal patients is well functioning (GOODCOOP)

##### Attitudes Towards Prevention

Four items with four response alternatives from ”Do not agree at all” to ”Agree completely” designed to measure the attitudes towards the possibility to prevent suicidal acts. These items have previously been found to form a good fitting measurement model [49].

- It is possible to prevent suicides (PREVSUI)
- It makes no difference what is done for suicidal patients—they succeed sooner or later anyway (NODIFF)
- If people really want to kill themselves, they will succeed in spite of receiving the best treatment (SUCCEED)
- Once people have made up their minds to commit suicide, you cannot stop them (CANNOTST)

The responses to all attitude items were scored using a Likert scale ranging from 1 (do not agree at all) to 4 (agree completely) and were summarized into an index. The values for the negative items were reversed, *i.e.*, the higher value the more positive attitudes. 

*Multiple linear regression* analyses, using SPSS version 22, were used to develop models for predicting the three different outcome variables: *Job clarity*, *Job confidence* and *Attitudes towards prevention*. The predictive value of the independent variables was compared in each analysis. Significance was set at 0.05.

### 2.3. Ethical Considerations

A letter of consent was distributed to all participants together with the questionnaire with information on the study and explaining that all publications of the results will be on aggregated data only. Sensitive data was not collected, and all participant responses were de-identified, thus no ethical approval was required for the study.

## 3. Results and Discussion

### 3.1. Results

#### 3.1.1. Dimensionality, Validity and Reliability of Attitude Items

Three measurement models aiming at measuring attitudes towards clarity in work with suicidal patients (*Job clarity*), attitudes towards confidence in work with suicidal patients (*Job confidence*) and attitudes towards the possibility to prevent suicides (*Attitudes towards prevention*) were tested in this study. 

Five items were supposed to measure *Job clarity* (see Method for the wording). However, the model did not fit the data. According to the modification indices, the items seemed to measure different subcomponents. When the item “Having irreconcilable demands made on me by different people in the ward” was excluded the four remaining items formed a model with a good fit (Table 1). 

The presumed measurement model of *Job clarity* consisted of four items (see Method for the wording). All four remained after testing the dimensionality (Table 1).

The third model contained four items that previously have been found to form a good fitting measurement model for *Attitudes towards prevention* [49]. In this study as well, all four items remained after testing the dimensionality (Table 1).

The mean values was 2.99 (range 1.50–4.00) for Job clarity; 2.95 (range 1.25–4.00) for *Job confidence and 3.20 (range 1.50–4.00) for Attitudes towards prevention*. 

**Table 1:** Validity and reliability for indicators in the concepts *Job clarity*, *Job confidence* and *Attitudes towards prevention.*

Concept (Latent Variable)	Indicator (Observed Variable)	Model Fit						Quality of Indicators	
		X^2^	Df	*p*-Value	RMSEA	*p*-Value	CFI	Validity	Reliability
*Job clarity*	Know	3.55	2	0.17	0.08	0.26	0.98	0.84	0.71
	Clear							0.70	0.48
	Diffview							0.33	0.11
	Lackinfo							0.77	0.59
*Job confidence*	Clearres	2.67	2	0.26	0.05	0.37	0.99	0.67	0.46
	Conf							0.71	0.50
	Allresp							0.56	0.31
	Goodcoop							0.87	0.75
*Attitudes towards prevention*	Prevsui	4.31	2	0.12	0.00	0.079	1.00	0.62	0.38
	Nodiff							0.89	0.79
	Succeed							0.46	0.21
	Cannotst							0.59	0.34

As depicted in Table 1, all three models had a reasonable good overall fit, which means that the overall congruency between the models and the observed data is acceptable. The overall fit is measured with Chi-square, Root Mean Square Error of Approximation (RMSEA) and Comparative Fit Index (CFI). According to Jöreskog & Sörbom [61,62] the *p*-value for χ^2^ should be higher than 0.05, RMSEA lower than 0.08 and CFI between 0 and 1. 

The local fit of the individual parameters in each model varies however. The validity estimate is the loading of the observed variable on the latent variable and the reliability is the proportion of variance in the indicator that is explained by the variables that directly affect it. In the model for *Job clarity* for instance “Not knowing what is expected of me” (0.84), “I get clear and good instructions” (0.70) and “I lack knowledge and information on what is important in work with suicidal patients” (0.77) have high loadings while “Different superiors have various views” (0.33) has a rather low loading which also is reflected in the low explained variance (0.11).

#### 3.1.2. Work Experience and the Perception of being Sufficiently Trained

The responses to the items concerning work experience and the perception of being sufficiently trained for work with suicidal patients are presented in Table 2.

**Table 2:** Work experience and the perception of being sufficiently trained, total number and percentages, *n* = 358.

Items		Frequency	Percentage
Working in psychiatry	<1 year	11	3.1
	1–5 years	51	14.2
	>5 years	295	82.4
	No answers	1	0.3
Working with suicidal patients the past 6 months	Each day	60	16.8
	At least each week	148	41.3
	At least each month	111	31.0
	Not at all	28	7.8
	No answers	11	3.1
Experienced patient suicide	Yes	215	60.1
	No	136	38.0
	No answers	7	2.0
Sufficient training	Yes	187	52.2
	No	125	35.8
	No answers	43	12.0

#### 3.1.3. Regression Models

All three regression models predicted the respective dependent variables significantly (*Job clarity* *p* < 0.001, *Job confidence* *p* < 0.01, *Attitudes towards prevention* *p* < 0.01). The only significant predictor in all three models was “perceived sufficient training” (*p* < 0.01, β = 0.559 for *Job clarity*; *p* < 0.01, β = 0.53 for *Job confidence*; *p* < 0.01, β = 0.191 for *Attitudes towards prevention*). Age was another significant predictor for *Job clarity* (*p* < 0.05, β = 0.134), while experience of patient suicide was a significant predictor for *Job confidence* (*p* < 0.05, β = 0.137). See Table 3, Table 4 and Table 5. Sufficient training was a predictor with significant impact on all attitudes: the more sufficient participants perceived their training, the more positive attitudes they had.

**Table 3:** Predictors for *Job clarity.*

Model	Unstandardized Coefficients	Standardized Coefficients	t	Sig.	95.0% Confidence Interval for B	
	B	Beta			Lower Bound	Upper Bound
Constant	2.258		11.655	0.000	1.877	2.639
Gender	−0.050	−0.046	−0.931	0.353	−0.156	0.056
Age	0.008	0.134	2.478	0.014	0.002	0.014
Time in psychiatry	−0.031	−0.028	−0.495	0.621	−0.153	0.091
Suicidal patients past 6 months	0.009	0.014	0.279	0.781	−0.054	0.072
Patient suicide	0.073	0.066	1.253	0.211	−0.042	0.188
Sufficient training	0.610	0.559	11.009	0.000	0.501	0.719

### 3.2. Discussion

The focus of this study was on attitudes surrounding working with suicidal patients and the preventability of suicidal acts, as these are considered important aspects regarding quality of care and the psychiatric staffs’ perceived ability of preventing suicidal acts among patients [15,16,17,18,19]. A number of attitude items have been tested using confirmative factor analyses to establish the dimensionality of the items. These tests resulted in three models measuring the concepts of clarity in work with suicidal patients (*Job clarity*), confidence in work with suicidal patients (*Job confidence*) and attitudes regarding the prevention of suicidal acts (*Attitudes towards prevention*) and the models have been found to be satisfactorily valid according to recommendations by Jöreskogs & Sörboms [61,62]. However, the cutoff values for various goodness of fit indexes are discussed and stricter rules might be recommended. Preliminary analyses of Hu & Bentler [63] suggests for instance that the cut off value for CFI should be close to 0.95 and for RMSEA close to 0.06, compared to between 0–1 and lower than 0.08 [61,62].

**Table 4:** Predictors for *Job confidence.*

Model	Unstandardized Coefficients	Standardized Coefficients	t	Sig.	95.0% Confidence Interval for B	
	B	Beta			Lower Bound	Upper Bound
(Constant)	2.397		11.437	0.000	1.984	2.809
Gender	0.027	0.026	0.470	0.639	−0.087	0.142
Age	0.000	0.006	0.093	0.926	−0.006	0.007
Time in psychiatry	0.107	0.099	1.602	0.110	−0.025	0.239
Suicidal patients past 6 months	−0.040	−0.065	−1.165	0.245	−0.108	0.028
Patient suicide	0.146	0.137	2.293	0.023	0.021	0.272
Sufficient training	0.374	0.353	6.300	0.000	0.257	0.491

**Table 5:** Predictors for *Attitudes towards prevention*.

Model	Unstandardized Coefficients	Standardized Coefficients	t	Sig.	95.0% Confidence Interval for B	
	B	Beta			Lower Bound	Upper Bound
(Constant)	3.569		16.364	0.000	3.139	3.998
Gender	−0.061	−0.061	−1.024	0.307	−0.178	0.056
Age	0.000	−0.006	−0.085	0.932	−0.007	0.007
Time in psychiatry	−0.099	−0.098	−1.437	0.152	−0.235	0.037
Suicidal patients past 6 months	−0.047	−0.079	−1.292	0.197	−0.118	0.024
Patient suicide	−0.095	−0.095	−1.452	0.148	−0.225	0.034
Sufficient training	0.191	0.191	3.103	0.002	0.070	0.312

The mean values of staff’s attitudes towards *Job clarity* (2.99, range 1.50–4.00), *Job confidence* (2.95, range 1.25–4.00) were relatively high and for *Attitudes towards prevention* (3.20, range 1.50–4.00) very high, indicating a positive view on all three attitude concepts.

The proportion (52.2%) of staff that consider themselves sufficiently trained for work with suicidal patients is higher in this study compared with earlier studies in psychiatric clinics in Stockholm. In a study by Samuelsson & Åsberg [46] with 191 psychiatric nursing staff, only 25% considered themselves sufficiently trained for this work and another study with a random sample of 300 psychiatric staff [64] 44.3%. 

The perception of being sufficiently trained is not an objective measure and no guarantee of the quality of staff’s encounter with patients, since individuals may misjudge their own competence [65]. It may nevertheless affect the care provided to suicidal patients, since it implies a sense of confidence in one’s own ability to perform the difficult task of caring for these patients.

In previous studies, *the perception of having sufficient training for work with suicidal patients* has been shown to increase significantly after suicide-preventive training [9,34,36,39,42,48,53,64,66]. In this study we found that this perception is also the main predictor for attitudes towards clarity and confidence in work with suicidal patients, as well as the possibility to prevent suicidal acts.

*The perception of having sufficient training for work with suicidal patients* were the only predictor for attitudes towards the possibility to prevent suicidal acts, whilst *age* were similarly a predictor for *Job clarity* (probably due to more experience), as was *having experienced more than one suicide among patients* for confidence in work with suicidal patients. 

Although, other factors were shown to have an impact on attitudes, the perception of being sufficiently trained seems to be the most consistent and most important. It was a consistent predictor for all three attitude concepts, more consistent and important than past work experience. Thus, attitudes, at least those that are measured in this study, are very much due to perceived knowledge, suggesting that attitudes towards work and prevention might be valid outcomes when evaluating suicide-preventive training. A strong correlation between the perception of being sufficiently trained for work with suicidal patients and clarity in this work has also previously been found in a Swedish study [58] where those who considered themselves to be sufficiently trained seemed to be able to trust their own knowledge and were less affected by unclear routines. 

However, one intriguing finding in this study is that having experienced at least one patient suicide was a predictor for confidence in work with suicidal patients. One might think that such an experience instead would produce more fear and uncertainty. Earlier studies have shown that patient suicides often evoke emotions of grief, fear of blame, embarrassment, self-doubt, inadequacy and shame [67,68,69,70,71,72]. On the other hand some beneficial outcomes have also been noted, such as learning from the experience [72,73], which might explain why the experience of patient suicides is a predictor for confidence in work also in this study. Another explanation could be that in the psychiatric clinic, where the study took place, a great importance has been attached to improving suicide-preventive routines and to offer psychological support after the death of a patient. This and other cultural factors may of course affect the generalizability of these findings. It should also be noted, that the attrition rate in this study is 30%. This group of non-respondents may in some way have deferred from the studied population. However, in comparison to other similar studies an attrition rate of 30% is very low [74].In future studies we will test whether the training in suicide prevention that was given to those who participated in the study presented in this paper had a positive effect on their attitudes towards work and the possibility to prevent suicidal behavior.

## 4. Conclusions 

Providing suicide preventive education to health-care staff is likely to improving their attitudes towards the preventability of suicide, improving clarity regarding their role in the care for suicidal patients, and their general confidence in their suicide preventive activities. These improvements are likely to contribute to the prevention of suicide in the health care setting. 
